# Metabolomic Analyses of Blood Plasma after Oral Administration of D-Glucosamine Hydrochloride to Dogs

**DOI:** 10.3390/md10081873

**Published:** 2012-08-22

**Authors:** Tomohiro Osaki, Kazuo Azuma, Seiji Kurozumi, Yoshimori Takamori, Takeshi Tsuka, Tomohiro Imagawa, Yoshiharu Okamoto, Saburo Minami

**Affiliations:** 1 Department of Veterinary Clinical Medicine, School of Veterinary Medicine, Tottori University, 4-101 Koyama-minami, Tottori 680-8553, Japan; Email: kazuazu85@yahoo.co.jp (K.A.); tsuka@muses.tottori-u.ac.jp (T.T.); imagawat@muses.tottori-u.ac.jp (T.I.); yokamoto@muses.tottori-u.ac.jp (Y.O.); minami@muses.tottori-u.ac.jp (S.M.); 2 Koyo Chemical Co. Ltd., 3-11-15 Iidabashi, Chiyodaku, Tokyo 102-0072, Japan; Email: kurozumi@koyo-chemical.co.jp (S.K.); takamori@koyo-chemical.co.jp (Y.T.)

**Keywords:** amino acid, dog, glucosamine hydrochloride, metabolomic analyses

## Abstract

D-Glucosamine hydrochloride (GlcN∙HCl) is an endogenous amino monosaccharide synthesized from glucose that is useful in the treatment of joint diseases in both humans and animals. The aim of this study was to examine amino acid metabolism in dogs after oral administration of GlcN∙HCl. Accelerated fumarate respiration and elevated plasma levels of lactic acid and alanine were observed after administration. These results suggest that oral administration of GlcN∙HCl induces anaerobic respiration and starvation in cells, and we hypothesize that these conditions promote cartilage regeneration. Further studies are required to evaluate the expression of transforming growth factor-beta (TGF-β).

## 1. Introduction

Glucosamine (GlcN) is a derivative of cellular glucose metabolism and is also a component of glycosaminoglycans, hyaluronic acid, and proteoglycans in the cartilage matrix that form the ends of bones. Exogenous GlcN primarily exists in two forms, D-GlcN hydrochloride (HCl) and GlcN sulfate [1]. Both forms are immediately ionized into GlcN, and therefore D-GlcN∙HCl and GlcN sulfate are largely redundant [2]. In Japan, GlcN sulfate is a medical agent but not used as a nutritional supplement. In the Glucosamine/Chondroitin Arthritis Intervention Trial (GAIT), GlcN∙HCl supplements were used, and based on that, we chose the same form for our study [3].

GlcN is useful for the treatment of joint diseases in both humans and animals [4,5]. The bioavailability of GlcN has been reported to be 26% in humans [6], 19% in rats [7], 12% in dogs [8], and 2%–6.1% in horses [9,10,11]. The maximum drug concentration time (Tmax) after intravenous injection of GlcN in rats was 2 h [12], while Tmax after oral administration in dogs was 1 h [13]. These results suggest species-specific differences in GlcN absorption and metabolism. 

Glycosaminoglycans (GAGs) and proteoglycans are found at high concentrations in cartilage. The results of our previous studies suggested that oral administration of GlcN∙HCl (1 g per head per day) led to regeneration of both GAGs and proteoglycans [14]. We also found that it protected cartilage in experimentally induced osteoarthritis by inhibition of type II collagen degradation and enhancement of type II collagen synthesis in articular cartilage [15]. 

In the GAIT study, GlcN was orally administered for 24 weeks [3]. However, there have been no studies investigating the relationship between long-term oral administration of amino monosaccharides and amino acid synthesis. The aim of this study was to examine the metabolism of amino acids in dogs after oral administration of GlcN∙HCl for five weeks. 

## 2. Results

### 2.1. Principal Component Analysis of the Metabolic Profiles of GlcN∙HCl

**Figure 1:**
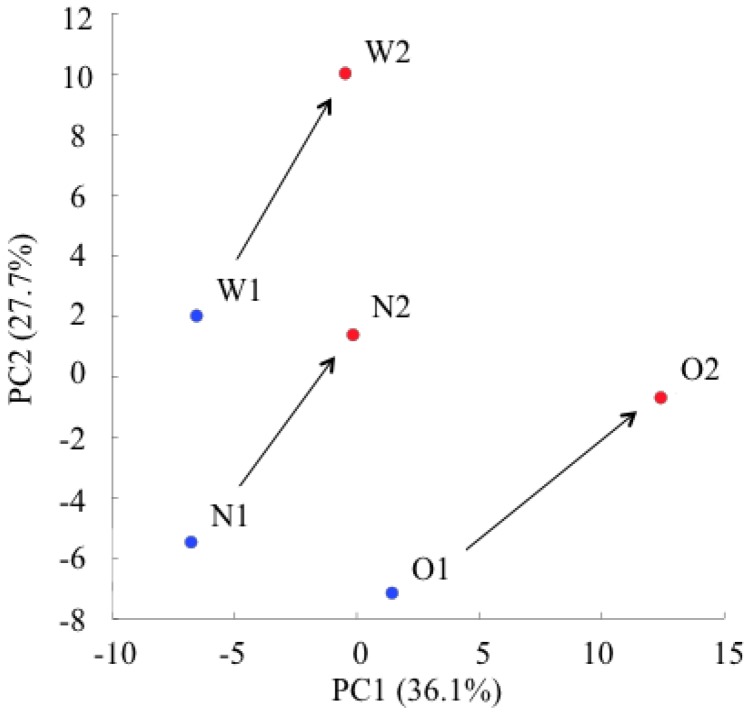
Principal component analysis (PCA) score plots before (blue dots: O1, N1, W1) and after (red dots: O2, N2, W2) oral administration of D-Glucosamine hydrochloride (GlcN∙HCl). Arrows indicate changing power directions after GlcN∙HCl administration in each dog.

A capillary electrophoresis time-of-flight mass spectrometry (CE-TOFMS) system detected 49 peaks (33 cation and 16 anion). Principal component analysis (PCA) is a technique for obtaining high-dimensional data that uses dependencies between variables to present data in a more tractable, lower-dimensional form, without losing too much information [16]. The PCA score plots of each dog changed in a similar manner before (O1, N1, W1) and after (O2, N2, W2) oral administration of GlcN∙HCl (Figure 1). The results revealed that the individual variability in the change in amino acid metabolism was small.

### 2.2. Changes in Plasma Metabolite Concentrations

**Figure 2:**
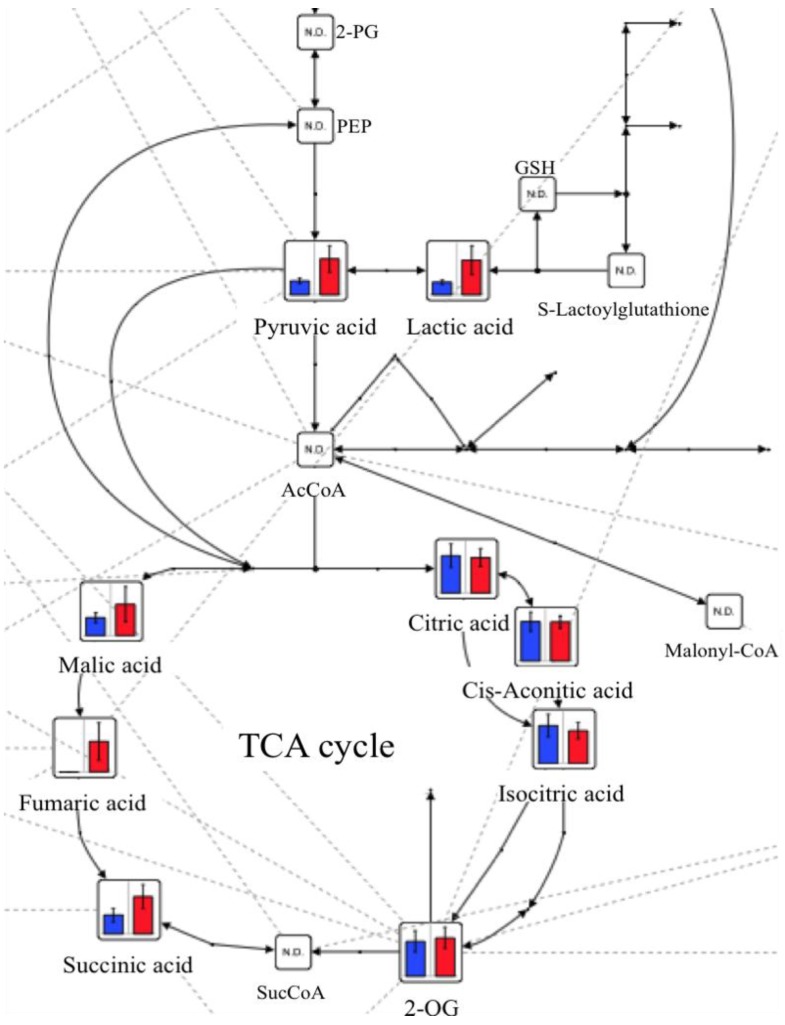
Levels of metabolites for glycolysis and the tricarboxylic acid (TCA) cycle before (**blue bars**) and after (**red bars**) oral administration of GlcN∙HCl.

Table 1 shows the concentrations of major metabolites before and after GlcN∙HCl administration. Glycolysis and the tricarboxylic acid (TCA) cycle are illustrated in Figure 2. The levels of succinic acid, malic acid, lactic acid, and pyruvic acid all increased after administration of GlcN∙HCl. Fumaric acid was not detectable before administration but reached a discernable level afterwards. The levels of hydroxyproline and alanine were significantly higher after oral administration of GlcN∙HCl than before (*p* < 0.01 and *p* < 0.05, respectively). 

**Table 1:** Concentrations (µM) of major metabolites before and after oral administration of D-Glucosamine hydrochloride (GlcN∙HCl).

Metabolites	Before		After	
	Mean	SD	Mean	SD
Glyoxylic acid	3.2	0.6	1.7	N.A.
Pyruvic acid	94	20	240	89
Lactic acid	1377	263	3760	1582
3-Hydroxybutyric acid	39	15	28	3.0
2-Hydroxybutyric acid	34	15	20	7.3
Fumaric acid	N.D.	N.A.	2.6	1.6
2-Oxoisovaleric acid	10	5.8	12	6.7
Succinic acid	7.4	2.8	15	4.8
Malic acid	7.6	2.1	13	7.6
2-Oxoglutaric acid	19	5.7	20	5.6
Glycerol 3-phosphate	2.8	0.5	4.0	1.1
*cis*-Aconitic acid	8.4	2.0	8.3	1.3
3-Phosphoglyceric acid	1.0	0.3	1.6	0.3
Citric acid	281	88	266	68
Isocitric acid	8.7	2.6	7.6	1.9
Gluconic acid	5.3	1.9	6.1	1.1
Glycine	144	8.5	148	25
Putrescine	0.6	0.06	0.7	0.13
Sarcosine *	3.8	0.6	5.9	0.3
Alanine *	291	42	417	39
β-Alanine	0.6	0.2	0.7	0.2
*N*,*N*-Dimethylglycine	4.9	0.9	6.1	1.6
Choline	6.3	2.9	9.5	2.2
Serine	109	14	132	36
Creatinine	48	4.7	45	3.8
Proline	87	16	127	26
Betaine	233	31	289	5.3
Valine	105	42	115	35
Threonine	116	28	150	65
Hydroxyproline **	3.7	0.11	5.7	0.3
Creatine	17	3.1	20	9.2
Isoleucine	36	13	41	5.8
Leucine	79	26	96	31
Asparagine	37	4.4	44	8.4
Ornithine	12	4.9	13	5.1
Aspartic acid **	4.1	0.3	6.4	0.5
Glutamine	755	177	907	204
Lysine	153	103	143	29
Glutamic acid	30	5.0	36	7.7
Methionine	29	4.0	34	2.4
Histidine *	79	3.5	93	6.9
Phenylalanine	53	8.7	56	6.4
Arginine	85	14	102	25
Citrulline	53	22	64	19
Tyrosine	37	5.8	44	6.6
Tryptophan	51	17	57	17
Carnosine	25	3.7	31	5.7
Cytidine	0.7	0.08	1.2	0.2
Glutathione (GSSG) divalent	0.6	0.09	0.8	0.3
Glucosamine	N.D.	N.A.	N.D.	N.A.
*N*-Acetylglucosamine	N.A.	N.A.	N.A.	N.A.

Asterisks indicate significant differences between the concentration of metabolites before and after oral administration of GlcN∙HCl. The *p* values were evaluated by the Welch’s *t* test. * *p* < 0.05; ** *p* < 0.01; SD: standard deviation; N.D.: not detected; N.A.: not available.

## 3. Discussion

Glucosamine is a widely used dietary supplement for promoting joint health. There have been concerns that oral GlcN supplementation at usual doses may adversely affect glucose metabolism in subjects with impaired glucose tolerance. However, a recent report showed that GlcN had no effects on fasting blood glucose levels, glucose metabolism, or insulin sensitivity at any oral dose in healthy subjects, in those with diabetes, and in those with impaired glucose tolerance [17].

In this study, GlcN was not detected in the plasma the day after stopping oral administration of GlcN∙HCl (day 36), which is in agreement with the results of our previous study [13]. That report revealed that amino acid levels in the plasma changed 1 h after oral GlcN∙HCl administration and that the metabolomics profile could change within a day. The observed changes in metabolomic profiles in the current study were definitely related to GlcN∙HCl administration because all other life cycle variables, including diet, were constant. Because the plasma levels of GlcN increased slightly just after feeding, we assume that GlcN did not denature. However, in the future we need to investigate the stability of GlcN∙HCl in food. 

In this study, levels of *N*-acetylglucosamine (GlcNAc) remained nearly unchanged before and after administration of GlcN∙HCl. Changes in GlcNAc levels were evaluated, but our measurement technique was not able to quantify its concentration in the plasma because of the lack of a standard substance. We assume that the administration of GlcN∙HCl did not affect the metabolism of GlcNAc. In future studies, the concentration of GlcNAc should be quantified using high performance liquid chromatography.

No prior reports have described the increases of succinic acid, malic acid, and fumaric acid resulting from oral administration of GlcN. It is known that some parasites and cancer cells depend on fumarate respiration using a reverse reaction involving succinate dehydrogenase, producing succinate as a byproduct [18,19]. Under conditions of glucose depletion and severe hypoxia, ATP is synthesized without oxygen by fumarate respiration. The active use of fumarate respiration after oral administration of GlcN∙HCl provides a possible explanation of the accumulation of fumaric acid observed in this study.

During glycolysis, the levels of lactic acid and pyruvic acid increased after administration of GlcN∙HCl. Lactic acid is a predominant source of carbon atoms for glucose synthesized by gluconeogenesis. Accumulated lactic acid is recycled by the liver via the Cori cycle to form glucose; however, this is an energy-consuming process. Yalamanchi *et al*. [20] showed that lactic acid significantly increased transforming growth factor-beta (TGF-β) peptide expression, receptor expression, and functional activity. TGF-β is a cytokine with numerous functions related to wound healing, including recruitment of fibroblasts and macrophages, stimulation of collagen production, and formation of new capillaries [21,22]. Cartilage regeneration might therefore result from its increase. 

During fasting, muscles break down protein, and amino acids are released into circulation. In the present study, the levels of hydroxyproline and alanine after oral administration of GlcN∙HCl were significantly higher than levels prior to administration, with alanine higher to a greater extent. Alanine can be directly converted to pyruvic acid and used as a substrate for gluconeogenesis (via the alanine cycle) [23], which could possibly explain the increase in plasma concentrations of pyruvic acid we found.

Glucose depletion might result from hexosamine-induced insulin resistance [24]. GlcN taken up by cells is phosphorylated into GlcN-6-phosphate by hexokinase, which then bypasses GFAT to enter the hexosamine pathway [25]. It is then converted to uridine diphosphate-GlcNAc. Elevated levels of GlcNAc inhibit insulin-stimulated glucose uptake (via GLUT4 translocation) and glycogen synthesis [24]. GlcNAc also serves as a donor substrate for the synthesis of nucleotides, glycosyl phosphatidylinositol anchors, complex protein glycosylation, and *O*-linked-β-*N*-acetylglucosamine (*O*-GlcNAc) protein modification. *O*-GlcNAc modification of cytosolic and nuclear proteins occurs via the action of *O*-GlcNAc transferase [24]. This post-translational modification enhances the transcription of TGF-β and plasminogen activator inhibitor-1 [24,26]. Similarly, TGF-β induced by *O*-GlcNAc might stimulate collagen production.

## 4. Materials and Methods

### 4.1. Chemicals

GlcN∙HCl was supplied by Koyo Chemical Co., Ltd. (Tokyo, Japan). 

### 4.2. Animals

Three healthy beagle dogs, mean age 6.3 years (range, 5–7 years) and mean body weight 10.4 kg (range, 9–11.1 kg), were used in this study. The use of these animals and all related procedures were approved by the Animal Research Committee of Tottori University. 

### 4.3. Administration and Blood Sampling

GlcN∙HCl (25 mg/kg) was mixed with standard dog food (Dog Meal; Cainz Home Co., Ltd., Gunma, Japan) just before feeding. The dose was set in proportion to the recommended human dose (1.5 g/65 kg). The dogs were fed at the same time daily for 35 days. Blood samples from each dog were collected before beginning GlcN∙HCl administration and again 36 days after. The samples were drawn from the jugular vein using heparin as an anti-coagulant. They were centrifuged at 734× *g* for 10 min, and the plasma was then immediately separated and frozen at −80 °C. 

### 4.4. Instrumentation

Capillary electrophoresis time-of-flight mass spectrometry (CE-TOF-MS) was carried out using an Agilent CE capillary electrophoresis system (Agilent Technologies, Waldbronn, Germany) equipped with an Agilent 6210 time-of-flight mass spectrometer, an Agilent 1100 isocratic HPLC pump, an Agilent G1603A CE-MS adapter kit, and an Agilent G1607A CE-ESI-MS sprayer kit. The overall system was controlled by Agilent G2201AA ChemStation software version B.03.01 for CE.

### 4.5. CE-TOFMS Conditions

Cationic metabolites were analyzed with a fused silica capillary column (50 µm i.d. × 80 cm total length) and commercial cation electrophoresis buffer (Solution ID: H3301-1001, Human Metabolome Technologies) as the electrolyte. Samples were injected at a pressure of 50 mbar for 10 s (approximately 10 nL), and the applied voltage was set at 27 kV. Electrospray ionization-mass spectrometry (ESI-MS) was conducted in the positive ion mode, and the capillary voltage was set at 4 kV. The spectrometer was scanned from *m/z* 50 to 1000. Other conditions were standard for cation analysis [27,28].

Anionic metabolites were similarly analyzed with a fused silica capillary column and commercial anion electrophoresis buffer (Solution ID: H3302-1021, Human Metabolome Technologies) as the electrolyte. Samples were injected at a pressure of 50 mbar for 25 s (approximately 25 nL), and the applied voltage was set at 30 kV. ESI-MS was conducted in the negative ion mode, and the capillary voltage was set at 3.5 kV. The spectrometer was scanned from *m/z* 50 to 1000. Other conditions were standard for anion analysis [28,29,30].

### 4.6. Data Analysis

Raw data obtained by CE-TOFMS were processed with MasterHands software [31]. Signal peaks corresponding to isotopomers, adduct ions, and other product ions of known metabolites were excluded from analysis. All signal peaks potentially corresponding to authentic compounds were extracted, and their migration times (MT) were normalized using internal standards (MetSul for cations and CSA for anions). The peaks were then aligned according to the *m/z* and normalized MT values. Finally, peak areas were normalized using the internal standards and by sample amount. Annotation tables were produced from CE-ESI-TOFMS measurements of standard compound and aligned with the datasets according to similar *m/z* and normalized MT values. The metabolic pathway map was provided using the public-domain software Visualization and Analysis of Networks containing Experimental Data (VANTED) [32,33].

### 4.7. Statistical Analysis

All values are presented as the mean ± standard deviation (SD). The Welch’s *t*-test was used to compare the amino acid concentrations in the plasma before and after oral administration of GlcN∙HCl. The results were considered significant at *p* < 0.05.

## 5. Conclusions

**Figure 3:**
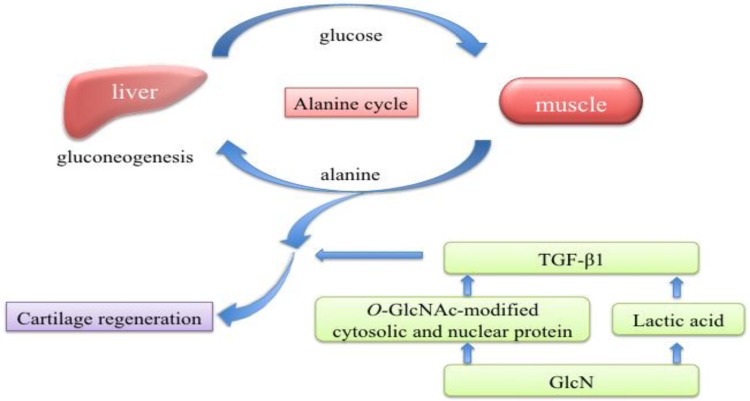
Schematic diagram of cartilage regeneration. Elevated plasma levels of lactic acid and alanine were observed after oral administration of GlcN∙HCl. We hypothesize that lactic acid and *O*-GlcNAc induce production of TGF-β, which would promote cartilage regeneration.

In this study, accelerated fumarate respiration and elevated plasma levels of lactic acid and alanine were observed after oral administration of GlcN∙HCl. These results indicate that oral administration of GlcN∙HCl induced anaerobic respiration and starvation in cells and further suggest that lactic acid and *O*-GlcNAc could potentially induce TGF-β production. We hypothesize that these conditions induced by oral administration of GlcN∙HCl promote cartilage regeneration as shown in the schematic diagram in Figure 3. However, further studies are required to evaluate the expression of TGF-β.
